# Application of A Precision Apiculture System to Monitor Honey Daily Production †

**DOI:** 10.3390/s20072012

**Published:** 2020-04-03

**Authors:** Pietro Catania, Mariangela Vallone

**Affiliations:** Department of Agricultural, Food and Forest Sciences, viale delle Scienze ed. 4, University of Palermo, 90128 Palermo, Italy; mariangela.vallone@unipa.it

**Keywords:** arduino, beekeeping, environment, hive, honey, precision agriculture, wind

## Abstract

Precision beekeeping or precision apiculture is an apiary management strategy based on the monitoring of individual bee colonies to minimize resource consumption and maximize the productivity of bees. Bees play a fundamental role in ensuring pollination; they can also be considered as indicators of the state of pollution and are used as bio monitors. Beekeeping needs continuous monitoring of the animals and can benefit from advanced intelligent ambiance technologies. The aim of this study was the design of a precision apiculture system (PAS) platform for monitoring and controlling the following environmental parameters: wind, temperature, and relative humidity inside and outside the hive, in order to assess their influence on honey production. PAS is based on an Arduino board with an Atmel microcontroller, and the connection of a load cell for recording the weight of the hive, relative humidity and temperature sensor inside the hive, and relative humidity and temperature sensor outside the hive using an anemometer. PAS was installed in common hives and placed in an open field in a French honeysuckle plot; the system was developed to operate in continuous mode, monitoring the period of 24 April–1 June 2019. Temperature was constant in the monitored period, around 35 °C, inside the hive, proving that no criticalities occurred regarding swarming or absconding. In the period between 24 and 28 May, a lack of honey production was recorded, attributed to a lowering of the external temperature. PAS was useful to point out the eventual reduction in honey production due to wind; several peaks of windiness exceeding 5 m s^−1^ were recorded, noting that honey production decreases with the peaks in wind. Therefore, the data recorded by PAS platform provided a valid decisional support to the operator. It can be implemented by inserting additional sensors for detecting other parameters, such as rain or sound.

## 1. Introduction

Precision agriculture (PA) is a subject of enormous interest for researchers. The goals of PA are to optimize crop production and to improve the quality of the final products; driving agriculture towards modernization [1], as agriculture is still a very traditional sector. The application of PA is also possible in a very important sector for the environment, such as beekeeping, giving rise to what is defined as precision beekeeping (PB) or precision apiculture, an apiary management strategy based on the monitoring of individual bee colonies to minimize resource consumption and maximize the productivity of bees [2].

The role of bees in ecology is linked to the pollination of profitable crops, but also to the conservation of spontaneous and wild crops, thus safeguarding a vast biodiversity. Bees also play an important role as indicators of the state of pollution and they are used as bio monitors in detecting and monitoring environmental pollution, particularly radioactive elements, pesticides, and toxic metals [3]. 

Beekeeping is an important part of agriculture, with distributed locations, needing the monitoring of animals in a 24/7 mode. The honey production cycle takes place inside beehives placed in an open field in the presence of plants in bloom. This depends on many factors, some of which are environmental factors, such as temperature, relative humidity, and wind [2,4], and can benefit from advanced intelligent ambiance technologies [5].

Many online data storage and real-time analysis systems have been recently developed using the PB approach in order to evaluate the hive internal parameters as temperature and relative humidity and the environmental condition such as temperature, relative humidity, rainfall, wind, and cloud cover. These parameters can be used to predict swarming or absconding processes having a strong relation with the health and productivity of beehive colonies [6]. 

A system to monitor honeybee colony temperature was developed in [7] using a wireless sensor networks where sensors nodes can communicate with a main module at a distance of 20 m in an open field. In [8], a prototype was developed to monitor and control bee colony activity in terms of weight of the beehive, outside and inside temperature, and humidity, which needed to be implemented in order to make it autonomous in terms of energy supply. The same parameters were measured by the system proposed in [9], which suggests the full integration of hardware to the hive structure for future research in order to facilitate beekeeper operations. All of these studies reaffirm that monitoring the environmental conditions of hives is the basis for improving their management and facilitating the work of beekeepers. They aim to add value to the apicultural chain, preserve the species, and take care of the environment. 

A bee swarm monitoring system, operating with audio signals captured in a beehive, is described in [5]; the captured beehive audio data contain different bee activities, based on different frequencies and energies of the signal. These were studied to carry out a bee swarm activity acoustic classification useful to improve beehive management.

The problem of radio frequency electromagnetic radiation (RF-EMR) emission, caused by the above-mentioned systems, was recently addressed in [6] to substantiate whether RF-EMR from Wi-Fi affects the stated in-hive measurements, by using a wired version of the developed sensor network. The authors concluded that there is no evidence of beehive environment change in response to RM-EMR, in the short term (30 days).

Wind is an apiary-located meteorological parameter useful to explain some peculiarities in colony behavior [10]. It is well known that low temperatures and wind are two factors that prevent bees from leaving the hive for foraging. The mating behavior of honey bee queens can be influenced by the presence of wind, particularly with high wind velocities (9–14 km/h) in combination with low temperatures (15–20 °C) [11]. Wind is considered one of the variables that can be assessed based on hive location. In [12] wind has been used to estimate the health status of bee colonies through the use of some classification algorithms, based on a supervised machine learning approach; the authors combined in-hive sensors data and external weather data to obtain information on the health status of a bee colony. Despite multiple references to the wind, the studies published so far do not show wind data directly in relation to the production of honey. 

In the two-year period of 2018–2019, particularly adverse weather conditions were recorded in Italy, with extreme events that were very frequent, intense, and harmful to beekeeping, causing considerable losses in production [13]. In various situations, there was a real zeroing of the honey production. Prolonged periods of drought, prolonged rainfall that damaged or canceled blooms, and low temperatures and wind, two factors that prevent bees from leaving the hive for foraging, are considered as extreme events. All these factors make it particularly important to implement monitoring strategies for hives that support their management by beekeepers.

The aim of this study was the application of a precision apiculture system (PAS) platform, previously designed by the authors [14], for monitoring and controlling the main environmental parameters, such as wind, temperature, and relative humidity inside and outside the hive, in order to assess their influence on daily honey production.

## 2. Materials and Methods

**Figure 1:**
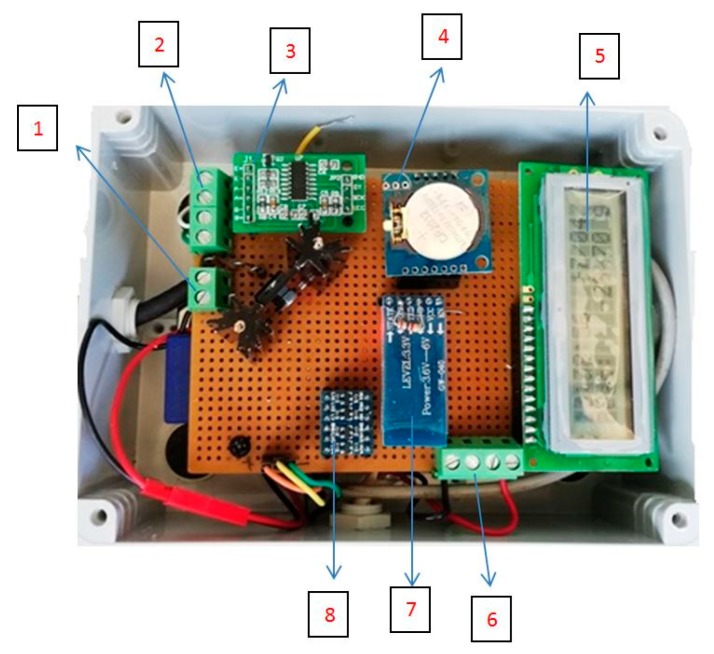
Arduino platform (1) external 12 V DC power supply connector; (2) load cell connector (and bridge power supply outputs and two bridge unbalance measurement inputs); (3) HX711 load cell amplifier; (4) real time clock DS1307; (5) Hitachi 16 × 2 display; (6) 12 V output for anemometer/vane power supply and two measurement inputs; (7) HC05 Bluetooth module; (8) level translator for SD card level converter 3.3 V/5 V.

The PAS was designed by the Department of Agricultural, Food and Forest Sciences of the University of Palermo and consists of an Arduino board with an Atmel microcontroller (ATmega2560) with 8 bits (Figure 1) The card has the following characteristics: operating voltage: 5 V, recommended input voltages: 7–12 V; maximum input voltage: 6–20 V: number of digital inputs/outputs: 54 (14 of which can be used as PWM outputs); number of analogue inputs: 16; current supported for input and output pins: 40 mA; current supported for 3.3 V pin: 50 mA; Flash memory: 256 KB (8 KB used for the bootloader); SRAM: 8 KB; EEPROM: 4 KB; clock frequency: 16 MHz. PAS is a platform capable of continuously recording and monitoring the following parameters: temperature (°C) and relative humidity (%) inside and outside the hive, quantity of honey produced (kg), and wind (m s^-1^).

The following sensors were connected to the card: load cell for recording the weight of the hive, relative humidity and temperature sensor inside the hive, relative humidity and temperature sensor outside the hive, and anemometer.

The metal load cell used was a model PSD-S1 with an analogue output signal, it has a degree of protection IP67, full-scale 100 kg, weight 551 g; linearity: ±0.02 (% F.S.); delay: ±0.02 (% F.S.); repeatability: ±0.02 (% F.S.); sensitivity: 2.0 mv/v, drift: ±0.02% F.S, input impedance: 350 ± 10 Ω, output impedance: 350 ± 5 Ω. 

The system was developed to operate in continuous mode. The two AM2302 relative humidity and temperature sensors used, one inside and one outside the hive, have dimensions: 3.7 × 2.1 cm, voltage: 5 V, temperature range: –40 – +80 °C +/− 0.5 °C, relative humidity range: 20–90% +/−2%. In particular, the internal sensor was placed in the nectar region to monitor the temperature of this environment, which is of fundamental importance for the activity of the queen [15]. These sensors allow to continuously record the daily environmental values of relative humidity and temperature. The objective was to compare the environmental values inside and outside the hive. As is known from the literature, the ideal temperature inside the hive for the production of honey must remain at 35 °C with a relative humidity of around 70%. 

The anemometer is equipped with a 3-arm cup rotor with solid-state magnetic sensor. It transforms the wind force into electrical signals. A shaft mounted on sliding bearings and fixed to the rotating vanes passes the magnet close to the contact. The impulses thus generated are transformed into an output voltage proportional to the wind speed. It has the following technical features: solid state transducer with frequency output; sensitivity: 3 km/h; precision: ±0.1 m/s; operating temperature: from 0 to 60 °C. The anemometer was placed near the hives.

The platform is also equipped with a removable SD card for data recording and subsequent download, and a Bluetooth module (HC05) which is connected to a common smartphone equipped with a Bluetooth serial application, allowing both the settings to change (time interval of the acquisitions) and the recorded data to automatically download.

The whole system was powered by two 100 A batteries alternately used to guarantee energy supply.

PAS has been subjected to verification tests in the laboratory in order to assess data reliability. Later, it was installed in common hives and placed in an open field for the first experimental tests that were performed in triplicate.

**Figure 2:**
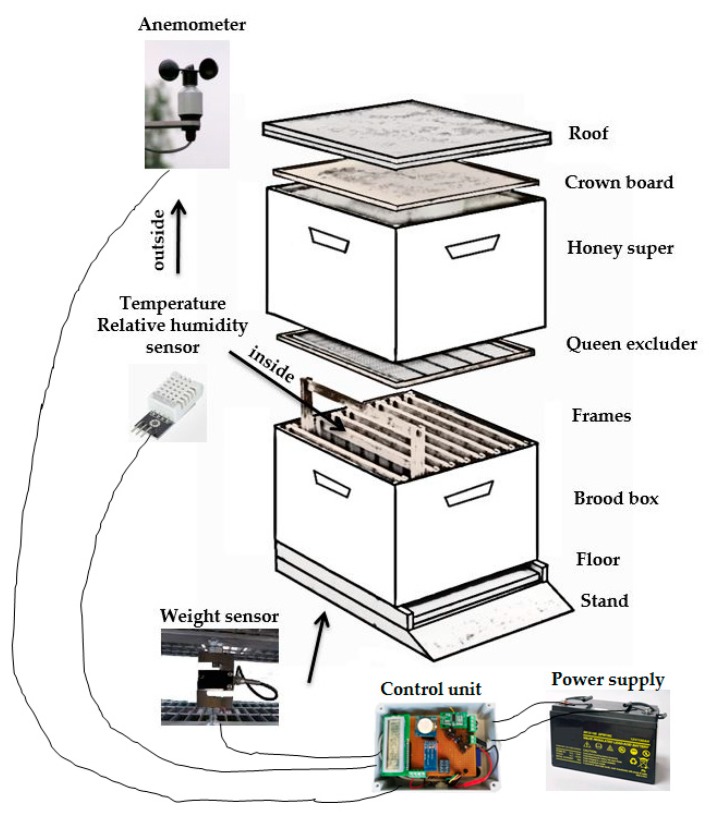
Layout of the devices used in the beehives.

PAS has been integrated into the hive consisting of the parts shown in Figure 2. The relative humidity and temperature sensor inside the hive has been inserted above the frames and under the queen excluder. The anemometer was placed in the center of the alignment of the hives near a row in the field. The weight sensor was positioned under the hive, inside a specially made metal structure.

**Figure 3:**
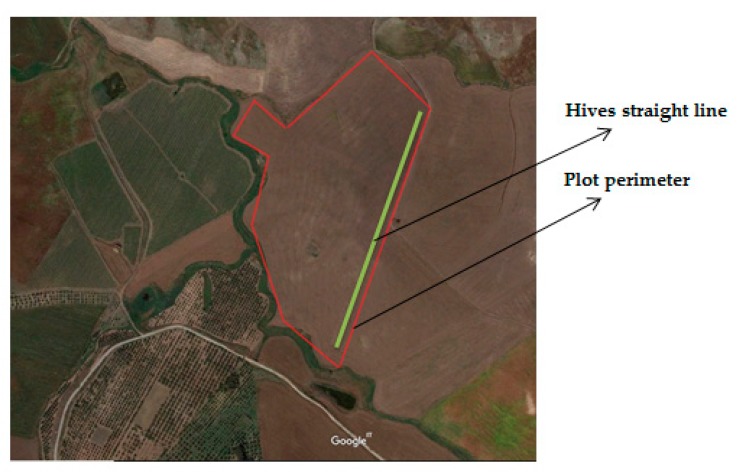
Experimental plot where the beehives equipped with the precision apiculture system (PAS) system were located.

The parcel chosen for the tests is located in the countryside of Monreale (Italy) Lat. 37°50′40.5″ N, Long. 13°03′11.0″ E. The plot, 260 m a.s.l., is approximately extended 20 hectares (Figure 3).

The species cultivated is a local variety of French honeysuckle (*Hedysarum coronarium L.*), one of the most important species for foraging in Mediterranean environments. It is a lively plant, normally biennial. The average height is about 1.50 m, the root is taproot, the stems have ribs varying in color from light green to dark red. The leaves are composed; the flowers are gathered in axillary purple-red racemes. Sowing took place in November 2018; the plant reached its maximum development in mid-spring coinciding with the flowering and lignification of the stems.

**Figure 4:**
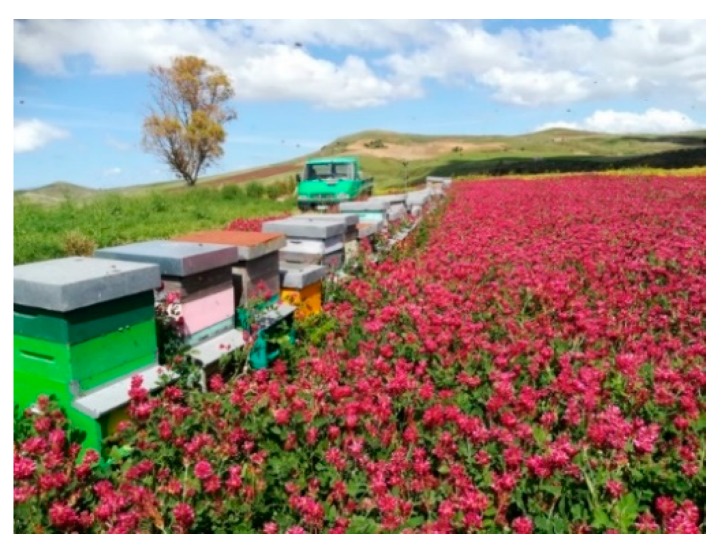
Hives in a French honeysuckle plot arranged along a straight line with an East–West orientation.

A total of 50 hives were placed, three of which were provided with the PAS platform. The hives were arranged at the beginning of the plot along a straight line with an East–West orientation (Figure 4).

Monitoring took place in the period between 24 April and 1 June 2019. Data collection was programmed for every 10 min.

Chemical–physical analyses were performed on a honey sample obtained during the test period in order to evaluate its quality. This was to confirm whether the honey production process was affected by any external factors, such as the choice of the site and the cultivated species, climatic trends, etc.

## 3. Results and Discussion

**Figure 5:**
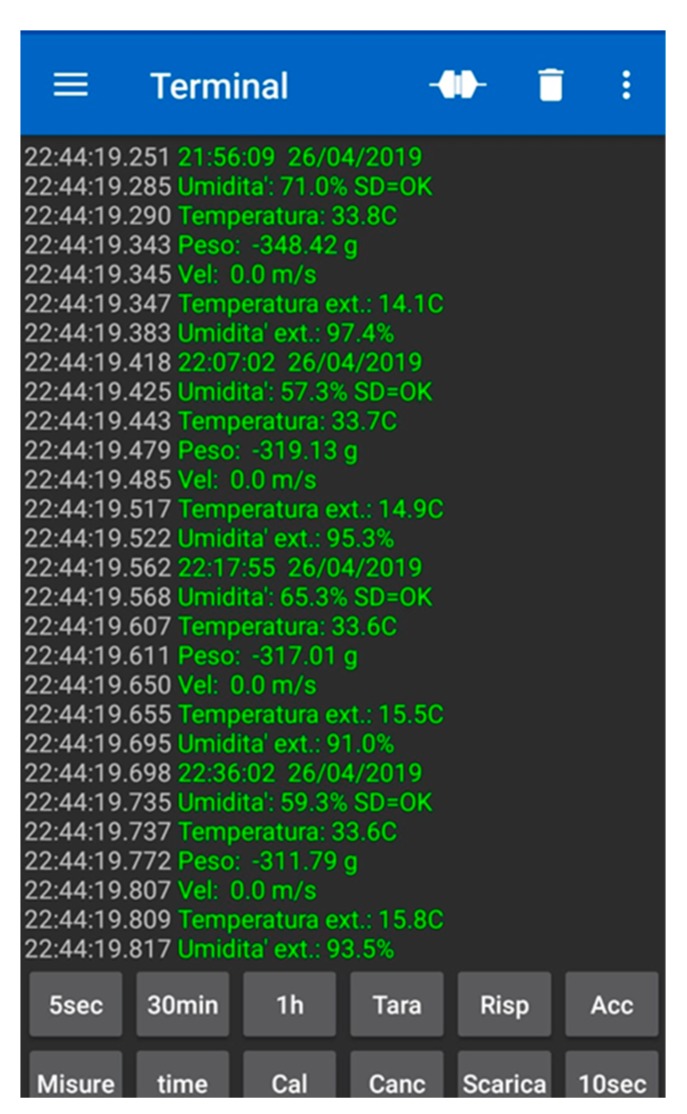
Example of a sheet screenshot with data downloaded from the PAS platform.

Data downloads were performed every seven days via an app on a common smartphone. A total of 4492 registrations were downloaded to a spreadsheet. A screenshot of the software during data download is shown in Figure 5.

**Figure 6:**
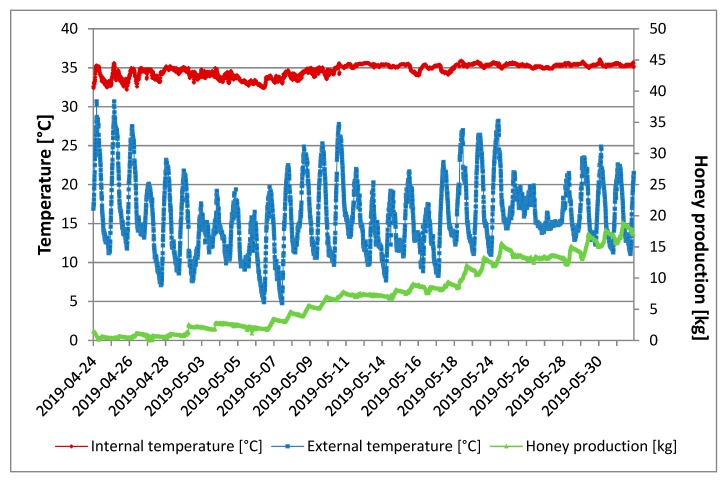
Daily quantity of honey produced as a function of internal and external temperature of the hive (data are the mean of three replicates).

Figure 6 shows the internal and external temperatures of the hive and the daily quantity of honey produced from the beginning to the end of the tests. The internal temperature remained almost constant (35 °C) for the entire period, both during the day and at night. A constant temperature of 35 °C inside the hive proved that, during the trial period, no criticalities occurred regarding swarming or absconding [16].

The outside temperature, on the other hand, showed a high variability; there were daily peaks of 30 °C in the first days of the tests and then peaks with lower values, but always above 20 °C. The temperature range between day and night was about 15 °C.

Honey production exceeded 5 kg after about 18 days from the placement of the hives in the field. 

During the whole monitoring period, there was a small decrease in honey production (about 200 g) during the night. This could be due to the fact that bees favor the evaporation of water from the nectar and pollen from day-foraging through the ventilation generated by the beating of wings [17].

In the periods of 1–6 May and 24–28 May there is a small decrease in honey production. The cause was attributed to a lowering of / external temperature, which was between 15 and 25 °C and thus the bees did not go out for foraging, and fed on the honey produced. In the same period, the internal temperature remained constant at about 35 °C.

In [18], a reduction of the internal temperature of the hive from 35 to 32 °C was obtained before swarming occurred. The authors stated that a possible explanation for this observation was the ventilation phenomenon, which consists of a rapid flitting of bees’ wings to reach a muscle temperature that is around 35 °C for lift-off [19]. This reduction was an important signal that if perceived by the beekeeper, would have avoided the loss of the bee colony. In our study, there were no significant reductions in internal temperature that allowed us to predict swarming. The slight reductions recorded can be attributed to the contemporary reduction of the external temperature.

**Figure 7:**
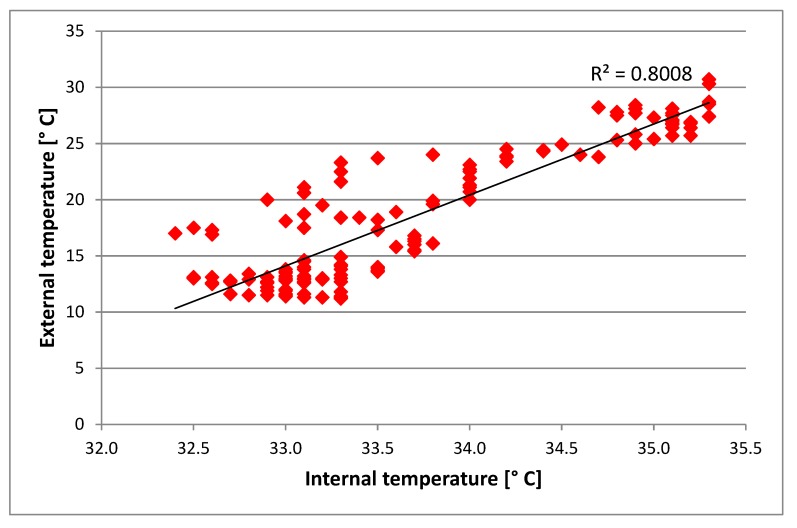
Correlation between external and internal temperature of the hives.

Figure 7 shows the correlation between internal and external temperature of the hives.

From the correlation of external and internal temperatures, we obtained r^2^ = 0.80, which is lower than the value found by Meikle et al. [20], equal to 0.90, and in [16], equal to 0.85. The difference was attributed to the sensor position inside the hive. In our study, the sensor was positioned at the center of the hive and precisely at the end of the nest, where the queen bee is found and the beginning of the honeycomb, where the formation of honey takes place. In [20], the sensor was placed in the upper part of the beehive and, therefore, closer to the external environment.

**Figure 8:**
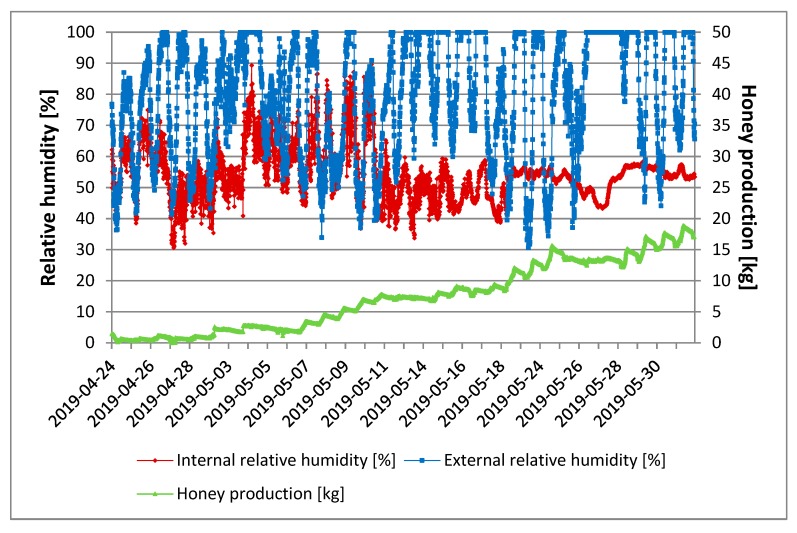
Daily quantity of honey produced as a function of internal and external relative humidity of the hive (data are the mean of three replicates).

Figure 8 shows that the honey production considerably increased from 11 May onwards, when the internal relative humidity did not exceed 60%. The external relative humidity in the same time period reached peaks close to 100% in the daytime hours. Relative humidity inside the hive decreased before swarming in [18]. The authors also reported that this could be due to the ventilation created by the bees.

In our study, in the period between 25 and 26 May, there was a decrease in internal relative humidity of about 10%. This reduction was not attributed to a possible swarming process as the external temperature also decreased. It should be noted that the internal temperature remained constant.

**Figure 9:**
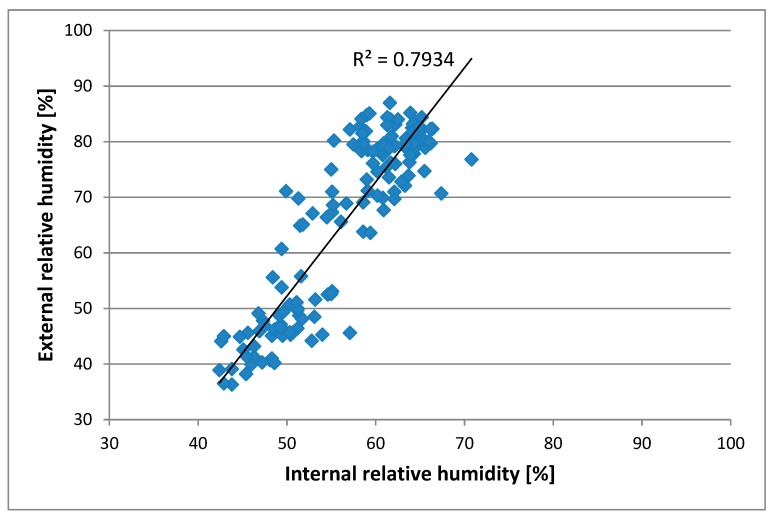
Correlation between external and internal relative humidity of the hives.

Figure 9 shows the correlation between external and internal relative humidity of the hives. There was also a high correlation (r^2^ = 0.79) for the relative humidity. This parameter was also in line with those obtained by other authors [20].

**Figure 10:**
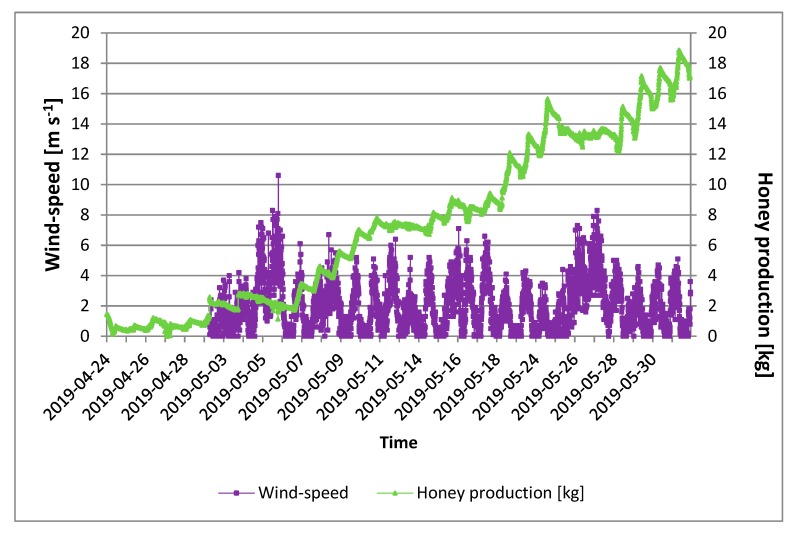
Daily quantity of honey produced as a function of external wind speed (data are the mean of three replicates).

During the monitoring period, several peaks of windiness exceeding 5 m s^−1^ were recorded. In only one case, we note the exceeding of 10 m s^−1^, which occurred on 6 May. It should be noted that honey production decreased with the peaks in wind. The decrease in honey production was greater the longer the windy period. Decreases in honey production were seen at wind peaks of over 4 m s^−1^ (Figure 10). In particular, this happened with greater evidence, for example in the periods 4–7 May and 25–28 May, 2019. It is clear that wind influenced the behavior of bees in terms of productivity. The reduction in honey was attributable to the lack of exit of the bees and their consumption of the product. Therefore, the choice of the site must provide for poor wind conditions, as it represents a strong environmental factor limiting the production of honey.

Regarding quality, honeysuckle honey is a quickly crystallizing type, and forms medium or fine crystals. When it is still in a liquid state, it has a color ranging from transparent to straw yellow, then turning to white–beige when crystallized. Crystallization is a natural process; it represents an index of quality in terms of authenticity, as honey is not subjected to industrial processes. Some types of honey, based on their compositions, do not tend to crystallize, while for others it can be a more or less fast process. Glucose and fructose are present in different concentrations in various types of honey. Glucose is less soluble than fructose in water; therefore, the higher the concentration of glucose, the greater the tendency to crystallize. Honey with more fructose, on the other hand, crystallizes more slowly. To assess the tendency to crystallization, the ratio of fructose to glucose is often considered, which must be less than 1.3 for slower crystallization. Table 1 shows the chemical–physical analyses performed on a honey sample that was taken from one of the beehives where a PAS was installed.

Chemical–physical analyses were performed according to the European Council Directive [21].

The honeysuckle honey produced during the PAS application revealed an excellent quality. The fructose and glucose ratio equaled 1.15 and the low presence of substances that are insoluble in water (0.1 g/100 g) allowed to obtain a slow and natural crystallization process, leaving unaltered the enzymatic and, therefore, nutraceutical properties of the product. The results of the analyses on honey attest that its qualitative parameters were within the limits of the law according to the above-mentioned European Council Directive.

## 4. Conclusions

This study made it possible to acquire information that was missing in the literature. The study of the main environmental parameters (temperatures, relative humidity, and wind speed) correlated with honey production and allowed it to manage the hive in an intelligent mode. 

The results obtained confirm the accuracy of the data recorded by the PAS platform, providing a valid decisional support to the operator (decision support system). This will make it possible to identify any critical points in the honey production process, namely: certain date of production start; daily production according to climate, certain date of production end; identification of the exact time to add/remove honeycombs, and warning of swarming. 

In years with adverse weather conditions, such as the one in which the tests were carried out (low temperatures and strong wind), during the bees’ foraging period, PAS could provide specific indications for beekeepers to minimize product losses. Finally, the authors will implement PAS by inserting other sensors for detecting rain and sound. PAS can allow to make key decisions that significantly improve both the health of the hive and the honey yield.

## Figures and Tables

**Table 1:** Chemical-physical analyses performed on a honey sample taken from one of the beehives where PAS was installed.

Parameter	Value	Limit Value
Lactone acidity (mEq/kg)	8.7 ± 4.0	
Free acidity (mEq/kg)	13.3 ± 6.7	50 mE/kg max
Total acidity (mEq/kg)	22.0 ± 7.8	
pH	3.7 ± 0.5	
Moisture (g/100 g)	16.7 ± 0.4	20 g/100 g max
Diastase (shade units/g)	12.1 ± 3.8	8 Shade units/g min
Fructose (g/100 g)	37.9 ± 1.1	
Glucose (g/100 g)	32.9 ± 1.0	
Fructose + glucose (g/100 g)	70.8 ± 1.5	60 g/100 g min
Sucrose (g/100 g)	1.6 ± 0.4	5 g/100 g max
Conductivity (mS/cm)	0.2 ± 0.01	0.8 mS/cm max
Hydroxymethylfurfural (mg/kg)	5.1 ± 1.3	40 mg/kg max
Substances insoluble in water (g/100 g)	0.02 ± 0.01	0.1 g/100 g max

Note: Data are the mean of three independent values ± standard deviation.
